# NURSE STAFFING AND VETERAN OUTCOMES IN THE VETERANS HEALTH ADMINISTRATION’S COMMUNITY LIVING CENTERS

**DOI:** 10.1093/geroni/igad104.2293

**Published:** 2023-12-21

**Authors:** Lana Brown, C Heath Gauss, Pamela Billings, Jade Moore, Linda Sawyer, Diane Sparks, Sheila Cox Sullivan

**Affiliations:** Central Arkansas Veterans Healthcare System, North Little Rock, Arkansas, United States; University of Arkansas for Medical Sciences, Little Rock, Arkansas, United States; Veterans Health Administration, Nashville, Tennessee, United States; Veterans Health Administration, Washington, District of Columbia, United States; Central Arkansas Veterans Healthcare System, North Little Rock, Arkansas, United States; Veterans Administration, Palo Alto, California, United States; Department of Veterans Affairs, Washington, District of Columbia, United States

## Abstract

Higher nurse staffing levels have been associated with better outcomes for patients in the acute care setting and for residents in the long-term care setting. There are several publications available linking nurse staffing to improved quality measures. Conversely, minimal research has occurred examining associations between nurse staffing and Veteran outcomes. The purpose of this research study was to assess for associations between nursing hours per patient day (NHPPD) and Veteran outcome measures in the Veterans Health Administration Community Living Centers. A retrospective data review was completed for NHPPD and quality measures for 134 Community Living Centers. Descriptive statistics were used to analyze the average total NHPPD for each of six Minimum Data Set, version 3.0, quality measures. Linear regression was utilized to assess for a linear association between average total NHPPD and the defined quality measures. This study found no linear association between average total NHPPD and the following Veteran outcome measures: improvement in function (p=0.15), ability to move independently worsened (p=0.13), catheter in bladder (p=0.48), and UTI (p=0.48). However, a statistically significant linear association was found between average total NHPPD and the following Veteran outcome measures: falls with major injury (p=0.02) and help with ADLs (p=0.01). As the average total NHPPD increased, the mean for falls with major injury and the mean for help with ADLs decreased. This study adds to the body of literature regarding the impact of nurse staffing on quality measures in the long-term care setting.

